# Case Report: Disseminated *Edwardsiella tarda* infection in an immunocompromised patient

**DOI:** 10.3389/fcimb.2023.1292768

**Published:** 2023-11-20

**Authors:** Lucia An, June L. Chan, Margaret Nguyen, Shangxin Yang, Jaime G. Deville

**Affiliations:** ^1^ University of California, Los Angeles (UCLA) Mattel Children’s Hospital, Department of Pediatrics, Division of Pediatric Hospital Medicine, Los Angeles, CA, United States; ^2^ University of California, Los Angeles (UCLA) Clinical Microbiology Laboratory, Department of Pathology and Laboratory Medicine, David Geffen School of Medicine, Los Angeles, CA, United States; ^3^ University of California, Los Angeles (UCLA) Mattel Children’s Hospital, Department of Pediatrics, Division of Pediatric Infectious Disease, Los Angeles, CA, United States

**Keywords:** *Edwardsiella tarda*, case report, immunocompromised, spontaneous bacterial peritonitis, disseminated infection

## Abstract

Human infection caused by bacteria of the *Edwardsiella* genus is rare and most often presents with gastroenteritis that rarely requires antibiotics. Our case report describes a medically complex patient with chronic steroid use contributing to an immunocompromised state, who presented with fever and abdominal pain. The patient was later found to have *Edwardsiella tarda (E. tarda)* bacteremia and underwent paracentesis confirming *E. tarda* bacterial peritonitis requiring a prolonged antibiotic course. This case report aims to illustrate the presentation, diagnosis, and management of an uncommon infection that can have severe complications especially among immunocompromised patients.

## 1 Introduction

Of the *Edwardsiella* genus, *E. tarda* is the most commonly isolated species and the main species known to cause disease in humans (J Michael Janda and Abbott, 1993). These motile, facultative anaerobic gram-negative rod-shaped bacteria are often found in fresh and brackish water environments and among wildlife including reptiles, amphibians, and fish (J MichaelJanda and Abbott, 1993). Human infection caused by *E*. *tarda* is rare with septicemia comprising only 5% of infections (J MichaelJanda and Abbott, 1993). However, septicemia is a severe complication and can be associated with a high mortality rate of up to 40-50% (Nelson et al., 2009). Our case demonstrates the successful management of *E. tarda* septicemia and bacterial peritonitis in which there is no standardized treatment protocol.

## 2 Case description

A 20-year-old female with a complex past medical history of hemophagocytic lymphohistiocytosis, macrophage activation syndrome, idiopathic thrombocytopenic purpura, systemic juvenile idiopathic arthritis, chronic lung disease, pulmonary hypertension, adrenal insufficiency, portal hypertension, and congestive hepatopathy presented with fever of 102 degrees Fahrenheit. She reported abdominal pain localized to her lower abdomen, worsening of chronic abdominal distention, decreased oral intake, and hematochezia.

The patient had been hospitalized 2 weeks prior for a hemorrhage complication after percutaneous liver biopsy that required hepatic embolization by interventional radiology (IR). She was also previously receiving immunosuppressive medications for immune thrombocytopenia and remained on stress dose steroids after discharge from the hospital.

On physical exam, the patient was well-appearing and not in acute distress. She had a cushingoid appearance with moist mucous membranes. Breath sounds were clear to auscultation, and she had no signs of increased work of breathing. Her abdomen was severely distended, soft, with diffuse mild tenderness to palpation, and with normoactive bowel sounds. She had resolving ecchymoses along the extremities from the prior admission and pitting edema in the lower extremities to the mid-shin.

## 3 Timeline

**Figure 1:**
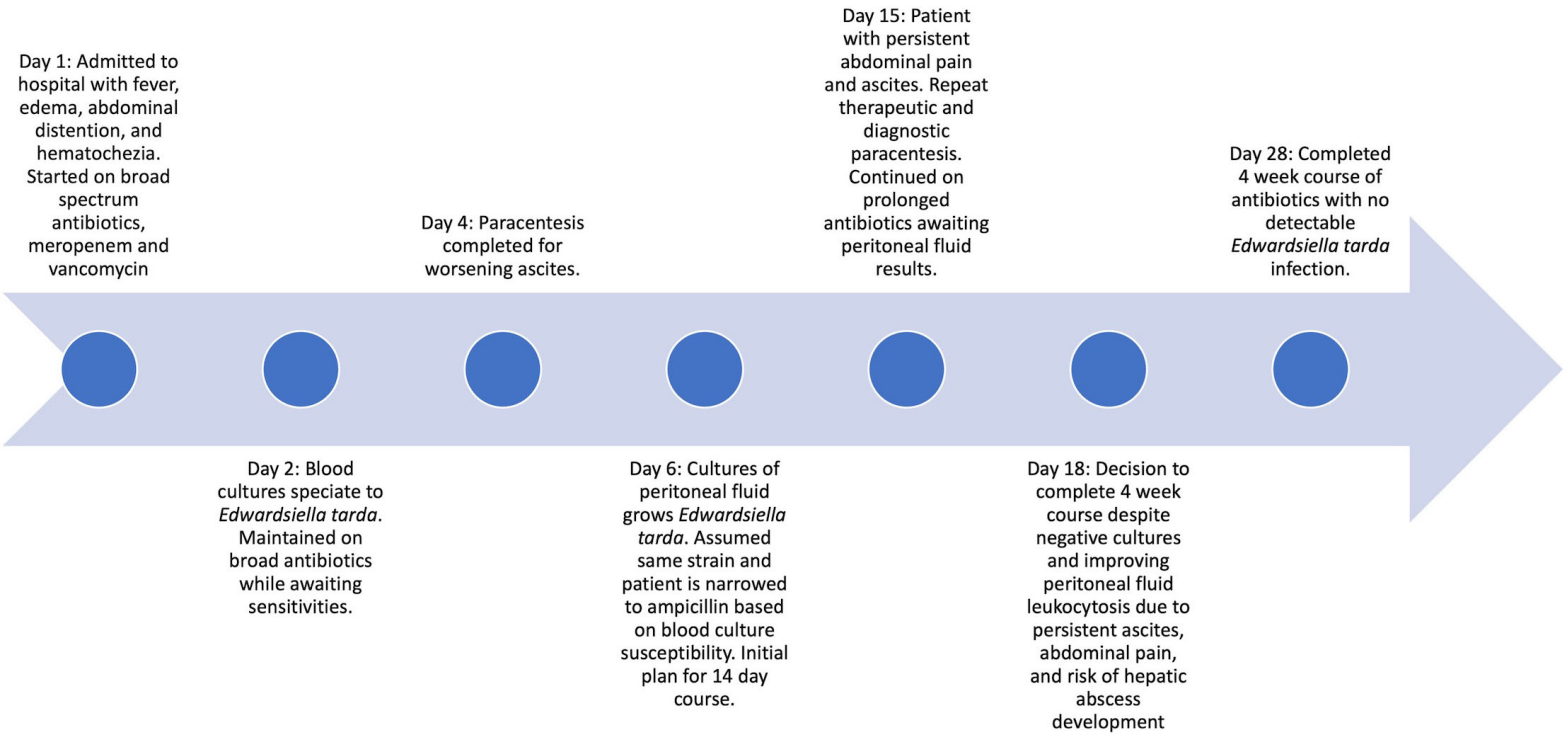
Treatment timeline.

The patient’s treatment course is illustrated in **Figure 1**.

## 4 Diagnostic assessment

In terms of laboratory testing, a complete blood count showed a white blood cell count 3.6x10^3^/uL, hemoglobin 11.1g/dL, and platelets 77 x10^3^/uL. A comprehensive metabolic panel showed alanine aminotransferase 28 U/L, aspartate aminotransferase 41 U/L, alkaline phosphatase 202 U/L, total bilirubin 3.6 mg/dL, direct bilirubin 2.8 mg/dL, total protein 4.6 g/dL, albumin 2.9 g/dL, lipase 37 U/L, lipase 37 U/L, INR 1.1, prothrombin time 14.2 seconds, c-reactive protein 0.9mg/dL, and lactate 17 mg/dL. Blood cultures were drawn prior to initial antibiotic dosing.

Diagnostic imaging included an abdominal x-ray with no significant colonic stool and hazy density overlying the abdomen suggestive of ascites; a chest x-ray showed no new consolidation or pleural effusion, persistent pulmonary vascular congestion, and stable enlarged cardio-mediastinal silhouette; a CT of abdomen and pelvis with and without contrast showed near complete resolution of the left upper quadrant hematoma seen on her previous admission, an evolving hemoperitoneum also seen in her previous admission but with no extravasation of contrast to suggest active bleeding, stable hepatic lesions seen on previous imaging; and an abdominal ultrasound (US) of the right upper quadrant showed moderate ascites and coarsened liver parenchyma with nodular contour, compatible with diffuse liver disease.

The initial differential was broad given the patient’s medical complexity and included bacteremia, recurrence of macrophage activation syndrome, a viral process in the presence of pancytopenia, and bacterial gastroenteritis given the history of hematochezia and abdominal pain. Spontaneous bacterial peritonitis (SBP) was also considered given the presence of ascites and fever.

### 4.1 Diagnostic evaluation and therapeutic intervention

The patient was empirically started on broad spectrum antibiotics, intravenous vancomycin and meropenem, given her medical complexity and immune-compromised state from underlying disease and stress dosed steroids. Blood cultures drawn at admission grew gram-negative rods. IR was consulted to perform a paracentesis for SBP given the recent abdominal procedure and presence of ascites. IR successfully drained 2.6L of amber-colored fluid with cell count studies showing a white blood cell count of 21,106 cells/mm3 with 80% segmented neutrophils, 13% lymphocytes, and 7% monocytes and cell chemistries showing 1.5g/dL protein, albumin 1.0 g/dL, 599 LD U/L, and 59 mg/dL glucose. The fluid studies were most concerning for SBP given the presence of leukocytosis with neutrophil predominance and lactate dehydrogenase elevation seen with infections.

Infectious disease was consulted and performed a thorough exposure history. This revealed that the patient had consumed raw fish and raw oysters 5 days prior to admission. This exposure was consistent with the bacterial organism ultimately recovered. Antibiotics were narrowed to ampicillin after phenotypic antimicrobial susceptibility testing (AST) of the bacterial isolate confirmed susceptibility to beta-lactam drugs from both the blood culture and peritoneal fluid culture.

### 4.2 Microbiology and genomic assessment

**Figure 2:**
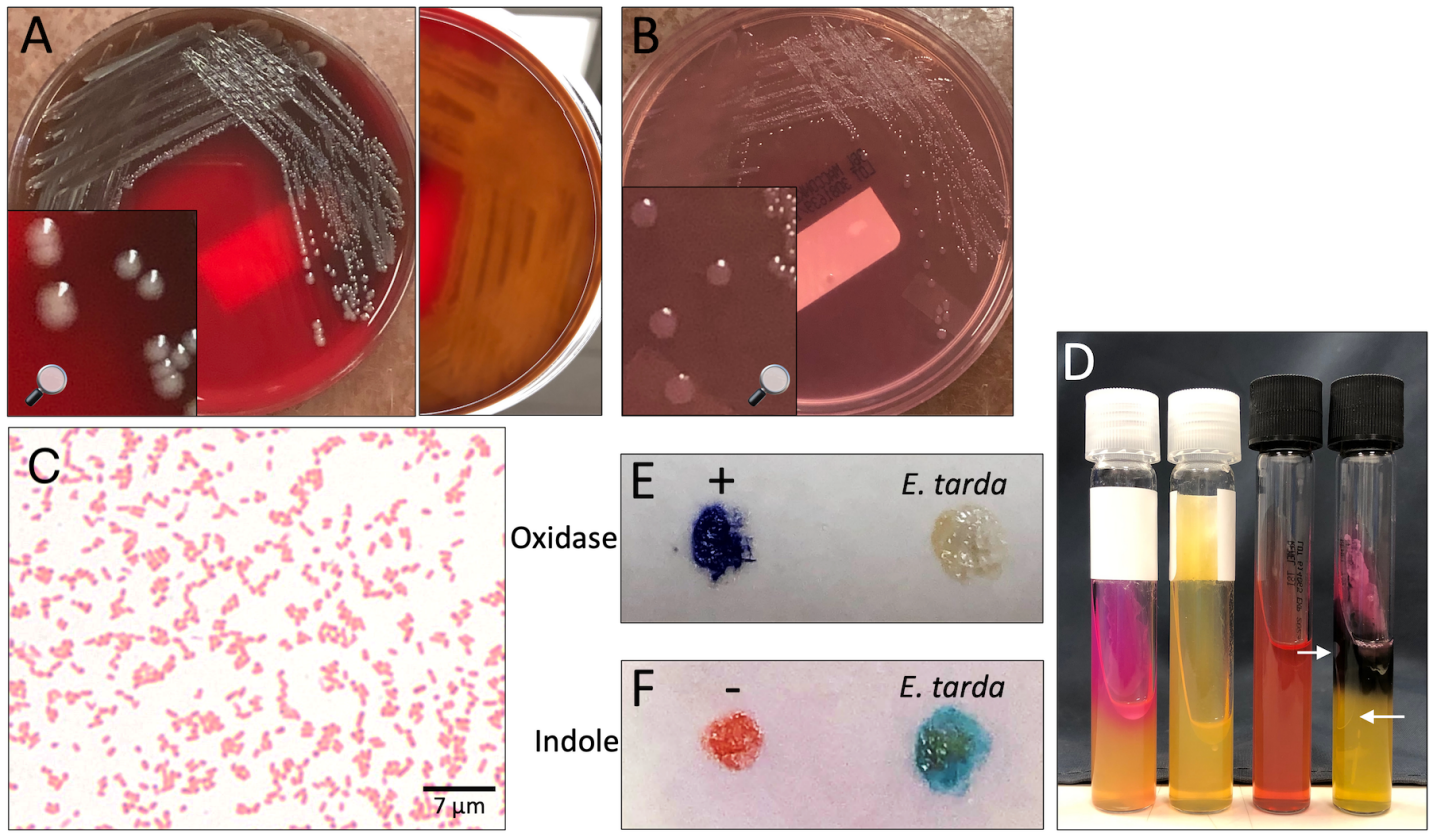
*Edwardsiella tarda* recovered from patient blood. **(A, B)** Isolate growth after 36 hours incubation. **(A)** Colonies on sheep blood agar. Colonies exhibit β-hemolysis with distinct zones of clearing on plate reverse. **(B)** Colonies on MacConkey agar. Colonies remain colorless due to inability to ferment lactose. **(C)** Gram stain showing straight gram-negative rods, 1000X magnification. **(D)** From left to right: positive urease control (*Klebsiella pneumoniae*), *E tarda* negative urease, uninoculated triple sugar iron (TSI) slant, *E tarda* TSI slant with K/A, Gas, H_2_S result. Arrows point out gas bubbles. **(E)** Spot oxidase test *E tarda* negative result (no color change to violet). **(F)** Spot indole test *E tarda* positive result (color change to turquoise-blue).

Two blood culture sets were obtained from the patient on day of presentation to the emergency department. Both aerobic and anaerobic bottles of each blood culture set turned positive within 15 hours of specimen loading to the blood culture monitoring system (BD BACTEC™ FX) with gram-negative rods seen on stain. The next day, the isolate was identified using MALDI-TOF mass spectrometry (bioMérieux, VITEK^®^ MS) and supplemental biochemical testing to confirm the bacterial isolate as *E. tarda* (**Figure 2**). A Gram stain of peritoneal fluid collected by paracentesis 4 days after initial presentation yielded many white blood cells with no bacteria seen. After 2 days of incubation, *E. tarda* was additionally recovered from the peritoneal fluid aerobic culture plates. The clinical presentation suggests that these isolates were the same strain from the same source. AST profiles of blood and peritoneal *E. tarda* isolates were identical (**Table 1**).

**Table 1:** Antimicrobial susceptibility testing of *E. tarda* isolated from bacterial blood culture and peritoneal fluid bacterial culture.

Source: Blood	Antibiotic	MIC (mcg/ml)	Interpretation
	Ampicillin	<= 0.5	Susceptible
	*Ampicillin + Sulbactam	<= 8	Susceptible
	Cefepime	<= 0.5	Susceptible
	Ciprofloxacin	<= 0.25	Susceptible
	Gentamicin	<= 1	Susceptible
	Piperacillin + Tazobactam	<= 8	Susceptible
	Trimethoprim/Sulfamethoxazole	<= 1/20	Susceptible
Source: Peritoneal fluid	Antimicrobial	MIC (mcg/ml)	Interpretation
	Ampicillin	<= 4	Susceptible
	Cefepime	<= 0.5	Susceptible
	Ciprofloxacin	<= 0.25	Susceptible
	Gentamicin	<= 1	Susceptible
	Piperacillin + Tazobactam	<= 8	Susceptible
	Trimethoprim/Sulfamethoxazole	<= 1/20	Susceptible

Antimicrobial susceptibility testing was performed by broth microdilution by the UCLA Clinical Microbiology Laboratory on custom antibiotic trays prepared in-house.

*Tested on blood isolate upon provider request.

To perform genomic characterization of the *E. tarda* blood isolate (NCBI Reference Sequence: SRR25388894), the DNA of the bacteria was extracted using the Qiagen EZ1 DNA Tissue Kit. The library was prepared using the Illumina Nextera DNA Library Preparation Kit, and sequencing was performed with the Illumina MiSeq using the 2X250bp protocol, which generated 3.7 million sequence reads. *De novo* assembly was performed using the Qiagen CLC Genomics Workbench v22.1 and the assembly sequence was uploaded to KmerFinder (Center for Genomic Epidemiology, https://cge.food.dtu.dk/services/KmerFinder/), which identified the patient’s *E. tarda* isolate to be most closely related to *E. tarda* strain KC-Pc-HB1 (NCBI Reference Sequence: NZ_CP023706.1); whole-genome mapping using the Biomatters Geneious Prime revealed the sequences of the patient’s isolate covered 96.2% of the reference genome, with 98.7% pairwise similarity, and a mean coverage/depth of 158.3X, indicating high genetic similarity. The patient’s *E. tarda* blood isolate was further categorized as *Edwardsiella* sequence type 6. Antimicrobial resistance (AMR) gene analysis was performed using the Qiagen CLC Genomics Workbench v22.1, which did not yield the presence of any significant AMR genes after querying the following databases: ResFinder (https://cge.food.dtu.dk/services/ResFinder/) and The Comprehensive Antibiotic Resistance Database (CARD, https://card.mcmaster.ca/). This finding was consistent with the pan-susceptible phenotypic AST profile of our isolate. The *E. tarda* KC-Pc-HB1 reference strain was initially isolated from the blood of a false killer whale (*Pseudorca crassidens*), a tropical and warm temperate dolphin species, that had been caught along the South Sea (Republic of Korea) (Lee et al., 2018).

### 4.3 Follow-up and outcomes

The course of the patient was complicated by persistent ascites thought most likely attributed to underlying liver disease. She required multiple therapeutic paracentesis procedures that showed improving leukocytosis. Given the risk of bacterial seeding into peritoneal fluid and the risk of developing abscesses, the patient completed an extended 4-week course of antibiotics despite early clearance of blood cultures and resolution of fever. Her repeat bacterial cultures from both blood and peritoneal fluid remained negative, which was reassuring for infection clearance. The patient was also advised to avoid future consumption of raw seafood given her underlying medical conditions and high infection risk.

## 5 Discussion

Formally a member of the *Enterobacteriaceae* family and now part of the *Hafniaceae* family based on taxonomic and phylogenetics studies, *E. tarda* is an anaerobic gram-negative bacteria that ferments glucose but is unable to ferment lactose, sucrose, and mannitol (Jordan, 1969; Janda and Abbott, 2021). It is biochemically similar to *Salmonella* and is oxidase-negative with the ability to produce hydrogen sulfide; a key differentiating factor is its ability to degrade tryptophan into indole (Sakazaki, 2015). *E. tarda* is known to cause infections in aquatic birds and cultured fish, as well as marine mammals (Prymak et al., 1988; Miniero Davies et al., 2018). Infections in fish are often systemic, leading to ascites, hernia, exophthalmia, and lesions of the internal organs. Edwardsiellosis of cultured fish has led to massive economic losses in the aquaculture industry worldwide, and vaccine development to prevent disease in fish is an active area of research (Xu and Zhang, 2014). *E. tarda* virulence has been attributed to production of virulence factors, including catalases and contact-dependent hemolysins (J. M. Janda and Abbott, 1993), ability to invade epithelial cells, and resistance to phagocytic killing (Leung et al., 2012).

*E. tarda* can cause both localized and systemic infections in humans. The highest incidence rates occur in humid and subtropical climates and are likely related to dietary habits including raw food consumption.(J Michael Janda and Abbott, 1993; Hirai et al., 2015) The most common presentation in infected individuals is gastroenteritis, which amounts to 83% of reported cases and often resolves without antibiotic treatment (J Michael Janda and Abbott, 1993). Occupational and recreational exposures to fresh and brackish water environments also serve as potential seeding events for *E. tarda*. Wounds sustained in freshwater lakes, such as lacerations to the foot (Vartian and Septimus, 1990) and puncture wounds from catfish spines (Murphey et al., 1992; Hirai et al., 2015), have led to serious *E. tarda* skin and soft tissue infections (SSTIs). Several case reports have further described localized extraintestinal *E. tarda* infections, including liver abscesses, other intra-abdominal abscesses, genitourinary infections, and central nervous system infection (Sachs, 1974; J Michael Janda and Abbott, 1993; Wang et al., 2005; Takeuchi et al., 2009; Golub et al., 2010; Hirai et al., 2015; Suzuki et al., 2018; Bakirova et al., 2020).

*E. tarda* septicemia is a rare and severe complication with a high mortality rate. It is thought that *E. tarda* septicemia first develops through initial colonization or infection of the gastrointestinal tract. Our patient had ingested raw fish and raw oysters 5 days prior to clinical presentation. The genomic similarity of the blood isolate recovered from our patient to an isolate originally recovered from a false killer whale further demonstrates the environmental niche of *E. tarda* and its link to aquatic and marine animals.

*E. tarda* septicemic presentations tend to occur in patients with underlying hepatobiliary disease, diabetes mellitus, malignancy, and iron overload states, such as sickle cell disease (J Michael Janda and Abbott, 1993; Wang et al., 2005). Healey and colleagues recently described a rapidly fatal case of *E. tarda* bacteremia in a patient with advanced lung cancer, pulmonary hypertension, hepatitis C virus, and liver cirrhosis, acquired after the ingestion of raw oysters a day prior to presentation (Healey et al., 2021). Our patient had portal hypertension and congestive hepatopathy that may have contributed to her presenting with bacteremia and SBP.

Molecular and immunochromatographic assays for *E. tarda* detection have been developed for use in the aquaculture industry (Savan et al., 2004; Xie et al., 2013; Liu et al., 2015). Yet, the clinical diagnosis of human *E. tarda* infection still relies on recovery of the pathogen from microbiological culture. *E. tarda* can be easily identified in the clinical microbiology laboratory, as the organism grows readily on routinely used laboratory culture media (5% sheep blood agar, MacConkey agar) and displays a characteristic biochemical profile: indole-positivity and hydrogen sulfide production. In addition to manual biochemical testing, species identification can occur through use of kit-based commercial systems (i.e., bioMérieux API 20E or Vitek GNI Plus card) or MALDI-TOF mass spectrometry. Recovery of *E. tarda* from fecal specimens can be aided by use of selective and differential media including Hektoen enteric (HE) agar and xylose-lysine-deoxycholate (XLD) agar.

Most *E. tarda* strains are known to be susceptible to commonly administered therapeutic agents. The empiric treatment for *E. tarda* can begin with antimicrobials that typically target gram negative organisms, and *in vitro* studies have shown susceptibility to beta-lactams, cephalosporins, aminoglycosides, and oxyquinolones (J Michael Janda and Abbott, 1993). Historically, the greatest resistance has been to polymyxin B and colistin (Reinhardt et al., 1985) and more recently, *E. tarda* has been shown to have resistance to macrolides, lincosamides, streptogramins, glycopeptides, rifampin, and fusidic acid (Stock and Wiedemann, 2001). *E. tarda* is known to express beta-lactamase; however, it is likely only expressed in low levels and has not been shown to confer resistance to beta-lactams (J Michael Janda and Abbott, 1993; Stock and Wiedemann, 2001). For this patient, she was started on meropenem given her medical complexity, a broad-spectrum empirical treatment, and was narrowed to ampicillin after susceptibilities returned (TABLE).

Immunocompetent patients with gastroenteritis secondary to *E. tarda* do not typically require antibiotics, though severe infections benefit from treatment. In the immunocompromised or patients with significant underlying disease who have extraintestinal disease, prognosis is related to the extent of infection and the ability for source control. For patients with *E. tarda* bacteremia, liver cirrhosis is an independent risk factor for death (Hirai et al., 2015). In the pediatric population, there have been a few reported cases of *E. tarda* infection in immunocompromised patients. These include cases of patients who had a renal transplant with acute gastroenteritis, Cushing’s syndrome secondary to adrenal gland hyperplasia with liver abscess and sepsis, Crohn’s disease with gastroenteritis and an inflammatory bowel disease flare, and X-linked chronic granulomatous disease with osteomyelitis (Spencer et al., 2008; Kawai et al., 2011; John et al., 2012; Li et al., 2019). Fortunately, though these patients had weakened immune systems from their disease state or from medications, they all recovered. For some, discontinuing immunosuppressive therapy or finding the source of immunosuppression facilitated recovery (Spencer et al., 2008; John et al., 2012). For our patient who was on stress dose hydrocortisone due to adrenal insufficiency, the steroids could not be discontinued, but were gradually weaned.

In conclusion, though an infection secondary to *E. tarda* is uncommon, the organism should be considered in patients with underlying illness and with food-borne or environmental exposures. For most immunocompetent patients, intestinal disease is common and does not frequently require antibiotic treatment. Conversely, for immunocompromised patients, extra-intestinal manifestations of the disease can occur, and initiating treatment with a beta-lactam will frequently provide sufficient empiric coverage.

## Data availability statement

The datasets presented in this study can be found in online repositories. The names of the repository/repositories and accession number(s) can be found below: https://www.ncbi.nlm.nih.gov/genbank/, SRR25388894.

## Ethics statement

Written informed consent was obtained from the individual(s) for the publication of any potentially identifiable images or data included in this article. Written informed consent was obtained from the participant/patient(s) for the publication of this case report.

## Author contributions

LA: Conceptualization, Investigation, Visualization, Writing – original draft, Writing – review & editing. JC: Conceptualization, Formal Analysis, Investigation, Visualization, Writing – review & editing. MN: Conceptualization, Investigation, Writing – original draft, Writing – review & editing. SY: Formal Analysis, Investigation, Supervision, Writing – review & editing. JD: Conceptualization, Supervision, Writing – review & editing.

## References

[B1] Bakirova G. H. Alharthy A. Corcione S. Aletreby W. T. Mady A. F. De Rosa F. G. . (2020). Fulminant septic shock due to Edwardsiella tarda infection associated with multiple liver abscesses: a case report and review of the literature. J. Med. Case Rep. 14 (1), 144. doi: 10.1186/s13256-020-02469-8 32900379 PMC7478901

[B2] Golub V. Kim A. C. Krol V. (2010). Surgical wound infection, tuboovarian abscess, and sepsis caused by Edwardsiella tarda: case reports and literature review. Infection 38 (6), 487–489. doi: 10.1007/s15010-010-0057-5 20931258

[B3] Healey K. D. Rifai S. M. Rifai A. O. Edmond M. Baker D. S. Rifai K. (2021). Edwardsiella tarda: A classic presentation of a rare fatal infection, with possible new background risk factors. Am. J. Case Rep. 22, e934347. doi: 10.12659/AJCR.934347 34873141 PMC8667629

[B4] Hirai Y. Asahata-Tago S. Ainoda Y. Fujita T. Kikuchi K. (2015). *Edwardsiella tarda* Bacteremia. A Rare but Fatal Water- and Foodborne Infection: Review of the Literature and Clinical Cases from a Single Centre. Can. J. Infect. Dis. Med. Microbiol. 26 (6), 313–318. doi: 10.1155/2015/702615 26744588 PMC4692300

[B5] Janda J. M. Abbott S. L. (2021). The changing face of the family enterobacteriaceae (Order: “Enterobacterales”): new members, taxonomic issues, geographic expansion, and new diseases and disease syndromes. Clin. Microbiol. Rev. 34 (2), e00174–e00120. doi: 10.1128/CMR.00174-20 33627443 PMC8262773

[B6] Janda J. M. Abbott S. L. (1993). Expression of an iron-regulated hemolysin by Edwardsiella tarda. FEMS Microbiol. Lett. 111 (2–3), 275–280. doi: 10.1111/j.1574-6968.1993.tb06398.x 8405937

[B7] Janda J.M. Abbott S. L. (1993). Infections associated with the genus edwardsiella: the role of edwardsiella tarda in human disease. Clin. Infect. Dis. 17 (4), 742–748. doi: 10.1093/clinids/17.4.742 8268359

[B8] John A. M. Prakash J. A. J. Simon E. G. Thomas N. (2012). Edwardsiella tarda sepsis with multiple liver abscesses in a patient with Cushing’s syndrome. Indian J. Med. Microbiol. 30 (3), 352–354. doi: 10.4103/0255-0857.99503 22885207

[B9] Jordan G. W. (1969). Human infection with edwardsieila tarda. Ann. Internal Med. 70 (2), 283. doi: 10.7326/0003-4819-70-2-283 5764505

[B10] Kawai T. Kusakabe H. Seki A. Kobayashi S. Onodera M. (2011). Osteomyelitis due to trimethoprim/sulfamethoxazole-resistant Edwardsiella tarda infection in a patient with X-linked chronic granulomatous disease. Infection 39 (2), 171–173. doi: 10.1007/s15010-011-0080-1 21246245

[B11] Lee K. Kim H. K. Park S. -K. Sohn H. Cho Y. Choi Y. -M. . (2018). First report of the occurrence and whole-genome characterization of Edwardsiella tarda in the false killer whale (Pseudorca crassidens). J. Vet. Med. Sci. 80 (6), 1041–1046. doi: 10.1292/jvms.17-0590 29695679 PMC6021894

[B12] Leung K. Y. Siame B. A. Tenkink B. J. Noort R. J. Mok Y. -K . (2012). Edwardsiella tarda - virulence mechanisms of an emerging gastroenteritis pathogen. Microbes Infect. 14 (1), 26–34. doi: 10.1016/j.micinf.2011.08.005 21924375

[B13] Li A. K. Barton M. Delport J. A. Ashok D. (2019). *Edwardsiella tarda* infection triggering acute relapse in pediatric Crohn’s disease. Case Rep. Infect. Dis. 2019, 1–3. doi: 10.1155/2019/2094372 PMC6446101 31016054

[B14] Liu H. Wang Y. Xiao J. Wang Q. Liu Q. Zhang Y. (2015). An immunochromatographic test strip for rapid detection of fish pathogen Edwardsiella tarda. Biores. Bioprocessing 2 (1), 20. doi: 10.1186/s40643-015-0047-7

[B15] Miniero Davies Y. Xavier de Oliveira M. G. Paulo Vieira Cunha M. Soares Franco L. Pulecio Santos S. L. Zanolli Moreno L. . (2018). Edwardsiella tarda outbreak affecting fishes and aquatic birds in Brazil. Vet. Q. 38 (1), 99–105. doi: 10.1080/01652176.2018.1540070 30668277 PMC6830998

[B16] Murphey D. K. Septimus E. J. Waagner D. C. (1992). Catfish-related injury and infection: report of two cases and review of the literature. Clin. Infect. Dis. 14 (3), 689–693. doi: 10.1093/clinids/14.3.689 1562661

[B17] Nelson J. J. Nelson C. A. Carter J. E. (2009). Extraintestinal manifestations of Edwardsiella tarda infection: a 10-year retrospective review. J. Louisiana State Med. Soc. 161 (2), 103–106. 19489391

[B18] Prymak C. McKee L. J. Goldschmidt M. H. Glickman L. T. (1988). Epidemiologic, clinical, pathologic, and prognostic characteristics of splenic hemangiosarcoms, (1985). J. Am. Vet. Med. Assoc. 193 (6), 706–712. 3192450

[B19] Reinhardt J. F. Fowlston S. Jones J. George W. L. (1985). Comparative in *vitro* activities of selected antimicrobial agents against Edwardsiella tarda. Antimicrobial. Agents Chemother. 27 (6), 966–967. doi: 10.1128/AAC.27.6.966 PMC180198 4026271

[B20] Sachs J. M. (1974). Sickle hemoglobinopathy and edwardsiella tarda meningitis. Arch. Pediatr. Adolesc. Med. 128 (3), 387. doi: 10.1001/archpedi.1974.02110280117018 4415769

[B21] Sakazaki R. (2015). “Edwardsiella,” in Bergey’s Manual of Systematics of Archaea and Bacteria. (Hoboken, NJ: John Wiley & Sons, Ltd), 1–12. doi: 10.1002/9781118960608.gbm01144

[B22] Savan R. Igarashi A. Matsuoka S. Sakai M. (2004). Sensitive and rapid detection of edwardsiellosis in fish by a loop-mediated isothermal amplification method. Appl. Environ. Microbiol. 70 (1), 621–624. doi: 10.1128/AEM.70.1.621-624.2004 14711699 PMC321279

[B23] Spencer J. D. Hastings M. C. Rye A. K. English B. K. Ault B. H. (2008). Gastroenteritis caused by *Edwardsiella tarda* in a pediatric renal transplant recipient. Pediatr. Transplant. 12 (2), 238–241. doi: 10.1111/j.1399-3046.2007.00869.x 18086238

[B24] Stock I. Wiedemann B. (2001). Natural Antibiotic Susceptibilities of *Edwardsiella tarda* , *E. ictaluri*, and *E. hoshinae* . Antimicrobial. Agents Chemother. 45 (8), 2245–2255. doi: 10.1128/AAC.45.8.2245-2255.2001 PMC90638 11451681

[B25] Suzuki K. Yanai M. Hayashi Y. Otsuka H. Kato K. Soma M. (2018). *Edwardsiella tarda* bacteremia with psoas and epidural abscess as a food-borne infection: A case report and literature review. Internal Med. 57 (6), 893–897. doi: 10.2169/internalmedicine.9314-17 29225255 PMC5891534

[B26] Takeuchi H. Fujita Y. Ogawa H. Shiomi K. Toyokawa T. Furukawa T. . (2009). Multiple brain abscesses in neonate caused by edwardsiella tarda -case report-: —Case report—. Neurol. medico-chirurgica 49 (2), 85–89. doi: 10.2176/nmc.49.85 19246871

[B27] Vartian C. V. Septimus E. J. (1990). Soft-tissue infection caused by Edwardsiella tarda and Aeromonas hydrophila. J. Infect. Dis. 161 (4), 816. doi: 10.1093/infdis/161.4.816 2319177

[B28] Wang I.-K. Kuo H. -L. Chen Y. -M Lin C. -L. Chang H. -Y. Chuang F. -R. . (2005). Extraintestinal manifestations of Edwardsiella tarda infection: Edwardsiella Tarda Extraintestinal Infection. Int. J. Clin. Pract. 59 (8), 917–921. doi: 10.1111/j.1742-1241.2005.00527.x 16033613

[B29] Xie G.-S. . (2013). Specific and rapid diagnosis of Edwardsiella tarda by a novel loop-mediated isothermal amplification targeting the upstream region of hlyb gene. J. Aquat. Anim. Health 25 (2), 110–118. doi: 10.1080/08997659.2013.781555 23639057

[B30] Xu T. Zhang X.-H. (2014). Edwardsiella tarda: an intriguing problem in aquaculture. Aquaculture 431, 129–135. doi: 10.1016/j.aquaculture.2013.12.001

